# Relationship between physical activity and latent profiles of regulatory emotional self-efficacy among high school students: a latent profile analysis

**DOI:** 10.3389/fpsyg.2026.1796256

**Published:** 2026-03-09

**Authors:** Hao Chen, Tianci Lu, Hanwen Chen, Lingzhi Wang, Jun Yan

**Affiliations:** 1 College of Physical Education, Yangzhou University, Yangzhou, Jiangsu, China; 2 Department of Physical Education, Nantong Tianjiabing Junior Middle School, Nantong, Jiangsu, China

**Keywords:** high school students, latent profile analysis, person-centered approach, physical activity, regulatory emotional self-efficacy

## Abstract

**Purpose:**

Regulatory Emotional Self-Efficacy (RESE) is defined as an individual’s confidence and self-assessment of his or her ability to regulate emotions, and is inextricably linked to daily life. To overcome the limitations of traditional dimensional analyses in revealing the overall combination of characteristics, the present study used latent profile analysis to explore the latent profile structure of RESE and to analyze its relationship with physical activity level and key demographic characteristics.

**Methods:**

Using latent profile analysis to explore the latent profiles of RESE. R3STEP analysis and multivariate logistic regression were used, respectively, to explore the associations of physical activity and demographic variables with the latent profiles of RESE.

**Results:**

RESE can be categorized into four latent profiles: Low Negative Regulatory Emotional Self-Efficacy (8.9%), Low Positive Regulatory Emotional Self-Efficacy (40.9%), Moderate Regulatory Emotional Self-Efficacy (30.0%), and Proficient Regulatory Emotional Self-Efficacy (20.2%). Follow-up analyses revealed that physical activity and gender were associated with RESE.

**Conclusion:**

(1) RESE identified four potential profiles in a population of high school students. (2) Gender was a significant predictor of the attribution of latent profiles of RESE. Females were more likely than males to fall into the Low Negative Regulatory Emotional Self-Efficacy group. (3) Physical activity is a significant predictor of RESE profiles. The lower the level of physical activity, the higher the likelihood of being classified into the two low Regulatory Emotional Self-Efficacy groups.

## 1 Introduction

High school represents a critical developmental transition characterized by intense academic pressure, complex interpersonal changes, and self-identity exploration. These challenges impose significant demands on students’ emotional experiences and regulation capabilities. Consequently, Regulatory Emotional Self-Efficacy (RESE), which refers to an individual’s confidence in managing both positive and negative affects, is paramount to the psychological adjustment and wellbeing of high school students (Chen et al., 2022; Zyberaj, 2022). High RESE serves as a vital protective resource; it not only facilitates stress management and academic performance but also reduces risks of depression and anxiety, ultimately fostering positive individual development (Ramos-Cejudo et al., 2024; De Castella et al., 2018). Therefore, exploring latent RESE profiles in high school students is essential for identifying at-risk groups, understanding individual differences, and designing targeted mental health interventions.

RESE is rooted in Bandura’s self-efficacy theory and refers to an individual’s level of confidence in his or her ability to effectively regulate his or her emotional state (Bandura, 1997). Rather than being unidimensional, this concept comprises three interrelated yet distinct components: self-efficacy in regulating positive emotions, involving the expression of joy or pride after success (Bandura et al., 2003); self-efficacy in regulating distress/frustration refers to an individual’s confidence in effectively managing emotions such as frustration and sadness triggered by setbacks and losses; and self-efficacy in regulating anger/rage focus on mitigating irritation and inhibiting impulsive behaviors (Caprara et al., 2008). Together, these three dimensions form the cornerstone of an individual’s sense of efficacy in emotion regulation. However, the traditional research paradigm focusing on total or mean scores of RESE across dimensions (i.e., the variable-centered approach) has significant limitations in its implicit assumption of group homogeneity. Focusing solely on linear associations with overall RESE scores may obscure the complex, heterogeneous patterns across the three dimensions (Zhang et al., 2019; Wang and Hanges, 2011). Ignoring such qualitative distinctions simplifies the complexity of emotion regulation, making it difficult to identify unique risks or tailor interventions. This ultimately weakens the study’s ecological validity and its ability to provide practical guidance. Consequently, adopting the person-centered approach of Latent Profile Analysis (LPA) is essential. By identifying subgroups with unique multidimensional profiles, this method allows for a precise examination of how these distinct patterns relate to key predictors and outcomes.

Following the identification of latent profiles of Regulatory Emotional Self-Efficacy (RESE), exploring their key determinants becomes a central step in understanding the mechanisms shaping distinct subgroups. Prior research indicates that individual characteristics, particularly gender, age, and family structure, serve as critical contextual factors shaping emotional development during adolescence (Xue et al., 2025; Huahua et al., 2022). Social Role Theory suggests that gender socialization imposes distinct emotional expectations. Specifically, societal norms often encourage emotional vulnerability in females, whereas they promote emotional restraint in males. Consequently, females tend to report lower efficacy in managing negative emotions compared to their male counterparts (Yuan et al., 2018; McRae et al., 2008). Consequently, gender is expected to be a salient predictor of RESE profile. Furthermore, family structure and age represent influential factors that cannot be overlooked; as the primary microsystem for individual development, the family environment often determines the availability of emotional support resources. Compared to those from intact families, students from blended or single-parent families may encounter greater emotional instability and resource scarcity, potentially undermining the foundation for developing regulatory emotional self-efficacy (Xue et al., 2025). Simultaneously, with increasing age, the academic and interpersonal challenges students face become increasingly complex, necessitating dynamic adjustments in their RESE (Silvers, 2022). Therefore, incorporating these demographic variables is essential for constructing a more comprehensive ‘risk portrait’ of high school students.

Beyond these relatively stable demographic characteristics, physical activity is recognized as a key predictor variable with high potential due to its wide range of physical and mental health benefits. Relevant studies have demonstrated that regular physical activity participation is not only significantly associated with improved overall mood status and reduced risk of depression and anxiety symptoms (Nie et al., 2025; Van Loo et al., 2023), but also strongly associated with self-efficacy (Tang et al., 2022; Kohl et al., 2012). The Conservation of Resources (COR) theory provides a robust framework for understanding how physical activity influences RESE (Hobfoll, 1989). Within this framework, physical activity functions as a vital psychosocial resource. Its participatory processes, such as skill mastery, and positive outcomes, like stress relief, directly reinforce an individual’s sense of mastery. This sense of mastery serves as the primary driver of self-efficacy formation (Bandura, 1997). Through the ‘gain spiral’ mechanism, these accumulated resources enhance an individual’s RESE (Yuan et al., 2018; Mu et al., 2024). Prior research has indicated that physical activity may have varying predictive effects on different aspects of emotion regulation, being particularly sensitive to specific weaknesses such as deficits in managing anger or distress (Tang et al., 2022). Therefore, adopting a person-centered approach is essential to clarify how activity levels influence unique combinations of emotional traits, providing a direct basis for designing precise, differentiated physical activity interventions for high school students.

In summary, adopting a person-centered approach allows for a more nuanced understanding of RESE by accounting for inherent heterogeneity among high school students. Moving beyond traditional variable-centered approaches, the present study utilizes Latent Profile Analysis (LPA) to identify distinct emotional self-efficacy patterns. Furthermore, we examine how physical activity and demographic variables predict profile membership to better understand the mechanisms underlying emotional inefficacy. Based on relevant theories and literature, we propose the following hypotheses:

*Hypothesis 1*: Regulatory Emotional Self-Efficacy (RESE) among high school students may exhibit latent heterogeneity, whereby several distinct latent profiles can be identified. These profiles may present differentiated patterns of positive and negative emotion regulatory efficacy, rather than merely reflecting differences in overall efficacy levels.

*Hypothesis 2*: Demographic characteristics (gender, age, family structure) may be differentially associated with RESE latent profile membership. In particular, gender may be significantly associated with membership in certain low-efficacy-related profiles.

*Hypothesis 3*: Physical activity levels may predict RESE latent profile membership. Compared to higher physical activity levels, lower physical activity levels may increase an individual’s likelihood of entering low-efficacy-related profiles, whereas higher physical activity levels may be associated with a greater probability of belonging to higher-efficacy-related profiles.

## 2 Research methods

### 2.1 Participants

Taking physical activity and RESE of high school students in Jiangsu Province as the research object. Stratified whole group sampling was used, and after the preliminary research, one high school in each of the four geographical cities and townships of Jiangsu Province, totaling eight high schools for high school students, was selected as the survey object. A total of 2,495 questionnaires were distributed through offline paper-and-pencil tests, and 2,269 valid data were obtained after invalid questionnaires were excluded, with a questionnaire validity rate of 90.94%.

### 2.2 Measures

#### 2.2.1 Demographic characteristics questionnaire

A self-administered questionnaire was used to obtain information on the demographic variables of the respondents, including age, gender, region, family structure, and other relevant information.

#### 2.2.2 International physical activity questionnaire-short form

The International Physical Activity Questionnaire (IPAQ) is divided into long and short forms, and this study utilized the short form of the Chinese version of the IPAQ introduced by Qu and Li (2004). The IPAQ has developed 3 levels of evaluation criteria (see Table 1) (World Health Organization, 2010). As part of the Integrated Health Assessment Questionnaire, it was used to assess the daily physical activity of the study participants, and it is a questionnaire that is widely used to measure physical activity in international and national contexts, and studies have shown that its reliability and validity are good.

**Table 1:** Physical activity level classification standards.

Clusters	Standard
High level of physical activity	Meet any one of the following two criteria: Engaging in vigorous-intensity physical activities on more than 3 days per week, with a total weekly physical activity level exceeding 1,500 MET-minutes. Engaging in a combination of walking, moderate-intensity, and vigorous-intensity physical activities on more than 7 days per week, with a total weekly physical activity level exceeding 3,000 MET-minutes.
Moderate level of physical activity	Meeting any one of the following three criteria: Engaging in vigorous-intensity physical activities for at least 20 min per day on more than 3 days per week. Engaging in moderate-intensity activities and/or walking for at least 30 min per day on more than 5 days per week. Engaging in any combination of walking, moderate-, and vigorous-intensity physical activities on more than 5 days per week, with a total weekly physical activity level exceeding 600 MET-minutes.
Low level of physical activity	Meeting either of the following two criteria: No reported physical activity. Some physical activity reported, but not sufficient to meet the criteria for the moderate or high physical activity categories.

The figure illustrates the level of physical activity of subjects assessed through the IPAQ questionnaire, categorized as high, medium or low, based on the number of days of activity and metabolic equivalents (MET-min/week) at different intensities.

#### 2.2.3 Regulatory emotional self-efficacy scale

The Regulatory Emotional Self-Efficacy Scale developed by Caprara and revised by Wen was used (Caprara et al., 2008; Wen et al., 2009). The scale consists of 12 questions with a 5-point scale containing three dimensions: positive emotion self-efficacy, despair self-efficacy, and anger self-efficacy. The higher the total score, the higher the degree of self-efficacy for emotion regulation. In this study, the Cronbach’s *α* for this scale was found to be 0.915.

### 2.3 Data analysis

First, SPSS 26.0 was used to organize and analyze the data for descriptive statistics of each variable. Then, Mplus 8.3 was used for latent profile analysis to classify the latent profiles of high school students’ RESE; Subsequently, a multinomial logistic regression analysis was conducted using SPSS 26.0 to examine the predictive effects of demographic characteristics on latent profile membership. In this model, the latent profile classification served as the categorical dependent variable, with the profile exhibiting the highest overall efficacy set as the reference category. Age, gender, and family structure were entered simultaneously as predictors to adjust for their mutual variance. This procedure allows for the evaluation of the unique contribution of each demographic variable while controlling for others. Results are reported as Odds Ratios (OR) with 95% Confidence Intervals (95% CI) to estimate the precision of the effects. Finally, the R3STEP method of the robust three-step approach was used to explore the predictive role of different levels (high, medium, and low) of physical activity on different latent profiles of RESE.

## 3 Results

### 3.1 Common method bias

Common method bias was assessed using Harman’s single-factor test. The results indicated that the variance explained by the first factor was 21.303% (<40%), suggesting no severe common method bias in this study.

### 3.2 Latent profile analysis of regulatory emotional self-efficacy among high school students

Prior to the latent profile analysis, descriptive statistics were calculated for all variables to examine the basic characteristics of the sample (see Supplementary Table S1 for details). To explore the latent profiles of RESE among high school students, a latent profile analysis was conducted based on participants’ scores. Models specifying 1 to 5 profiles were estimated, with fit indices summarized in Table 2. Generally, lower values of AIC, BIC, and aBIC indicate better model fit, while entropy closer to 1 suggests clearer latent profile separation and higher classification accuracy. Significant *p*-values for LMR and BLRT tests demonstrate that the model fit of profile k is significantly better than that of *k-1*. Additionally, all latent profiles should exceed 5% of the sample size (Wen et al., 2023). The results in Table 2 show that as the profiles increase, the values of the model fit metrics AIC, BIC, and aBIC keep decreasing, and the Entropy values keep increasing, indicating more accurate classification. Considering that the sample size of a single profile in the five-profile model is too small, accounting for only 2% of the total population, the four-profile model for RESE is selected as the best model for high school students. Beyond statistical fit indices, the selection of the four-profile solution was grounded in its theoretical interpretability and practical relevance. Comparisons revealed that the three-profile solution primarily captured ‘level effects’ (classifying students into generalized High, Moderate, and Low efficacy), thereby masking the heterogeneity of emotional regulation patterns. In contrast, the four-profile solution identified domain-specific deficits by distinguishing between students struggling with positive emotions and those struggling with negative emotions. This distinction is theoretically supported by Caprara’s framework, which posits that positive and negative regulation are independent mechanisms (Caprara et al., 2008). Practically, distinguishing these subgroups is vital for intervention. Specifically, low efficacy in managing negative emotions is predominantly associated with anxiety and distress (Bandura et al., 2003), whereas deficits in positive emotion regulation (akin to anhedonia) represent a distinct risk pathway for depression (Ely et al., 2021). Lumping these students into a single “low efficacy” group would mask these specific needs. Therefore, the four-profile model offers the most ecologically valid representation of the data.

**Table 2:** Fit information for latent profile analysis of regulatory emotional self-efficacy.

Number of profiles	AIC	BIC	aBIC	LMR	BLRT	Entropy	Group size
1	36,648.564	36,682.927	36,663.864	/	/	/	1
2	34,857.109	34,914.380	34,882.609	<0.001	<0.001	0.768	0.65/0.35
3	34,150.443	34,230.622	34,186.142	<0.001	<0.001	0.844	0.11/0.64/0.25
4	33,478.427	33,581.515	33,524.326	<0.001	<0.001	0.893	0.09/0.40/0.30/0.20
5	33,047.982	33,173.978	33,104.081	0.188	<0.001	0.932	0.12/0.38/0.02/0.20/0.28

**Figure 1:**
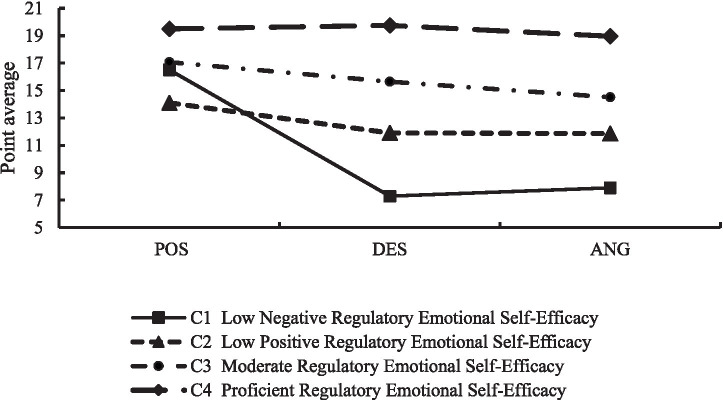
Means of scores on three factors for four latent categories of regulatory emotional self-efficacy. POS = perceived self-efficacy in expressing positive affect; DES = perceived self-efficacy in managing despondency/distress; ANG = perceived self-efficacy in managing anger/irritation.

The proportions of individuals in each level of RESE were 8.9, 40.9, 30.0, 20.2%. The scores on the three dimensions of self-efficacy in regulating positive emotions, self-efficacy in regulating frustration/distress emotions, and self-efficacy in regulating anger/irritability emotions are shown in Figure 1. The conditional means of the four latent profiles differed significantly across dimensions, revealing different characteristics. The mean values of C1’s self-efficacy in regulating frustration/distress and anger/irritability were lower than those of the other three groups, indicating that this group has a low sense of self-efficacy in negative emotion regulation and weak confidence in self-regulation, therefore, this group was named “Low Negative Regulatory Emotional Self-Efficacy (Low Negative RESE).” C2 has the lowest mean score on self-efficacy in regulating positive emotions, which indicates that this group has low efficacy in regulating positive emotions, so it is named “Low Positive Regulatory Emotional Self-Efficacy (Low Positive RESE).” The scores of C3 in each dimension are close to the overall mean, indicating that this group has a moderate and balanced sense of emotion regulation efficacy, so it is named “Moderate Regulatory Emotional Self-Efficacy (Moderate RESE).” C4 scored higher than the other three groups in all three dimensions, indicating that this group has a high sense of efficacy in positive and negative emotion regulation, so it was named “Proficient Regulatory Emotional Self-Efficacy (Proficient RESE).”

### 3.3 Demographic characteristics of latent profiles of regulatory emotional self-efficacy among high school students

To investigate the predictive effects of demographic characteristics on RESE profiles, a multinomial logistic regression analysis was performed. Latent profile membership served as the dependent variable, with the “Proficient RESE” profile set as the reference group. Age, gender, and family structure were specified as predictors to evaluate their unique contributions. Table 3 presents the detailed results, including unstandardized coefficients (B), standard errors (SE), odds ratios (OR), and 95% confidence intervals (95% CI). As presented in Table 3, gender emerged as a significant predictor of latent profile membership. Specifically, compared to females, males were significantly less likely to be categorized into C1 (Low Negative RESE), indicating that females exhibited a higher predisposition toward this profile. In contrast, age and family structure did not show statistically significant associations with any of the identified latent profiles (C1–C4), suggesting that these demographic factors do not differentiate high school students’ emotion regulation self-efficacy profiles in this sample.

**Table 3:** Multinomial logistic regression analysis of demographic variables on regulatory emotional self-efficacy.

Variable	Category	Low negative RESE				Low positive RESE				Moderate RESE			
		B	SE	OR	95% CI	B	SE	OR	95% CI	B	SE	OR	95% CI
Age		0.134	0.158	1.143	0.838–1.559	−0.018	0.106	0.982	0.797–1.209	0.103	0.112	1.108	0.890–1.380
Gender	Male	−0.920***	0.270	0.398	0.235–0.676	−0.084	0.176	0.920	0.651–1.300	−0.120	0.186	0.887	0.616–1.276
	Female	Ref.				Ref.				Ref.			
Family types	Intact family	−0.606	0.394	0.546	0.252–1.180	−0.151	0.309	0.860	0.470–1.574	0.074	0.332	1.077	0.561–2.067
	Blended family	0.064	0.574	1.066	0.346–3.287	−0.082	0.456	0.921	0.377–2.251	−0.403	0.514	0.668	0.244–1.829
	Single-parent family	Ref.				Ref.				Ref.			

Ref = reference group; Reference profile: proficient RESE. *p < 0.05; **p < 0.01; ***p < 0.001.

### 3.4 The predictive role of physical activity on regulatory emotional self-efficacy in high school students

To investigate the relationship between physical activity and RESE in high school students, this study employed the R3STEP method within the robust three-step approach. Analyses were conducted using latent profile analysis (LPA) results as the outcome variable and three-level physical activity (low, moderate, high; with high level as the reference group) as the predictor. As shown in Table 4, low physical activity significantly predicted classification into the Low Negative RESE group, the Low Positive RESE group, and the Moderate RESE group. Compared to the Proficient RESE group, students with low physical activity were more likely to belong to these groups than their peers with high physical activity, with odds ratios of 3.381, 2.127, and 1.779, respectively. For students with moderate physical activity, compared to the Proficient RESE group, they were significantly more likely to belong to the Low Negative RESE group (OR = 1.782) and the Low Positive RESE group (OR = 1.461), with no significant difference in their likelihood of belonging to the Moderate RESE group compared to high-activity peers. Collectively, these results establish a clear dose–response gradient between physical activity levels and RESE profiles. Lower physical activity consistently predicted elevated risks of classification into both low self-efficacy classes, with physical inactivity demonstrating the strongest negative association.

**Table 4:** The predictive role of physical activity on latent profiles of regulatory emotional self-efficacy (R3STEP analysis).

Variables	Low negative RESE				Low positive RESE				Moderate RESE			
	B	SE	OR	95% CI	B	SE	OR	95% CI	B	SE	OR	95% CI
Low physical activity	1.128**	0.390	3.381	1.576–7.256	0.755**	0.244	2.127	1.319–3.429	0.576*	0.249	1.779	1.092–2.899
Moderate physical activity	0.578*	0.293	1.782	1.003–3.166	0.379*	0.173	1.461	1.042–2.049	0.231	0.177	1.260	0.890–1.783
High physical activity	Ref.				Ref.				Ref.			

Ref = reference group; Reference profile: proficient RESE. *p < 0.05; **p < 0.01; ***p < 0.001.

## 4 Discussion

### 4.1 Latent profiles of regulatory emotional self-efficacy in high school students

This study used an individual-centered perspective and latent profile analysis (LPA) to explore the heterogeneous characteristics of high school students’ RESE. The results of the analyses indicate that there are significant internal differences in RESE among the high school student population, which is not a simple “high-low” continuum (Zhang et al., 2019), but can be clearly classified into four latent profiles with different characteristics. Among them, the “Low Negative RESE” group was the smallest (8.9%), with significantly lower scores than the other profiles on the dimensions of self-efficacy for regulating frustration/distress and self-efficacy for regulating anger/irritability. It indicates that they generally find it difficult to effectively manage and regulate their emotions when facing negative situations such as frustration, sadness or anger, and are prone to emotional distress and slow to recover (Bandura et al., 2003). The group with the highest proportion is the “Low Positive RESE” group (40.9%). They scored significantly lower on the dimension of self-efficacy in regulating positive emotions compared to the other three groups, indicating that these students have difficulties in experiencing, maintaining, and expressing positive emotions (such as joy, excitement, and pride). This may manifest as an inability to fully experience happiness, easily becoming “disappointed,” or struggling to maintain a positive mindset in moments of success. The high proportion of this group may stem from multiple factors, such as the neglect or suppression of positive emotional experiences in the current academic pressure environment, concerns about “extreme joy begetting sorrow,” a lack of effective training in positive emotion regulation strategies, or individual traits that are more inclined toward caution or the suppression of positive expressions, suggesting that the development of positive emotion regulation skills is a prevalent and important aspect of the current emotional education of high school students (Huang, 2018; Schall et al., 2016). The “Moderate RESE” group accounts for 20.0% of the sample. Their scores on the dimensions of self-efficacy in regulating frustration/pain, anger/irritability, and positive emotions are all close to the sample average, without showing any significant strengths or weaknesses in these areas. This suggests that such students hold a relatively “moderate” appraisal of their own emotion regulation capabilities, demonstrating consistent and stable levels of self-efficacy across diverse emotional contexts. They exhibit neither overconfidence nor excessive self-doubt, representing a group with a steady developmental trajectory in emotional competence. This suggests that this group maintains a relatively balanced level of confidence in managing both positive and negative emotions. In summary, the four latent profiles identified in this study not only methodologically validate the feasibility of a person-centered approach in regulatory emotional self-efficacy (RESE) research, but also provide a more nuanced perspective for understanding population differences in adolescent emotional development. Distinct from previous research approaches that treated RESE as a single continuous variable, this study identified a significant differentiation between the “Low Positive” and “Low Negative” profiles. This indicates that individuals’ self-efficacy in regulating positive and negative emotions may not change synchronously, but rather may be driven by relatively independent mechanisms. These results suggest that during adolescence, the development of emotion regulation capacities may exhibit dimensional imbalances, rather than simple overall high-low differences. Although this study employs a cross-sectional design, its findings provide a theoretical basis for further exploring the distinct pathways of positive and negative emotion regulation. It offers new insights into the developmental differences in high school students’ emotion regulation capacities and lays the groundwork for implementing more targeted interventions in the future.

### 4.2 Demographic characteristics of the latent profiles of high school students’ regulatory emotional self-efficacy

Multinomial logistic regression analysis indicates that gender serves as a significant predictor of the latent profiles of high school students’ RESE. These results partially supported Hypothesis 2. In terms of gender, compared to the “Proficient RESE” group, males were significantly less likely than females to be associated with the “Low Negative RESE” profile. In other words, females demonstrated a significantly higher likelihood of being classified into the Low Negative RESE group compared to males, a finding consistent with previous research (Chen et al., 2022; Eagly and Wood, 2013). This disparity may primarily stem from differences in gender role expectations during the socialization process. Previous literature indicates that females are often entrusted with the responsibility of caring for others’ emotions and maintaining interpersonal harmony (Huahua et al., 2022). These social norms may frequently expose females to negative emotional experiences, requiring them to invest greater energy in perceiving and managing emotions. However, during the high school stage, the dual pressures of academic competition and interpersonal relationships may exacerbate this emotional burden, potentially leading to the excessive depletion of emotional resources (McRae et al., 2008). Such chronic resource depletion might undermine an individual’s confidence in their ability to successfully regulate negative emotions, thereby increasing the likelihood of females being classified into the “Low Negative RESE” profile. In contrast, sociocultural norms typically encourage males to exhibit “toughness” and “restraint.” These norms may prompt males to report higher levels of control or efficacy in subjective assessments to align with societal expectations of masculinity (Yuan et al., 2018). Although this study did not directly examine these specific social factors, when viewed in conjunction with prior research, differences in gender role expectations offer a plausible theoretical perspective for interpreting the findings observed in this study. Neither age nor family structure had a significant predictive association with the membership of latent profiles of RESE. This may be because the study was conducted with high school students, who are at a relatively stable stage of psychological development in late adolescence, making it difficult to reflect the short-term changes associated with increasing age. As a result, no major differences were observed in age. In terms of family structure, the cross-sectional design of this study makes it difficult to capture the cumulative effects of changes in family structure (e.g., adaptation in reorganized families, length of single-parent families, etc.). In addition, high school students may buffer the influence of family structural differences on their perceptions of emotion regulation efficacy through external systems such as school and peer communities. As a result, their individual dependence on family support systems may be weakened. Taken together, the results also suggest a need to focus on developing females’ emotion regulation skills—particularly by helping them enhance their self-efficacy in coping with negative emotions and in overcoming the additional pressures imposed by social expectations.

### 4.3 Relationship between physical activity level and latent profiles of regulatory emotional self-efficacy in high school students

Our findings reveal that high school students’ RESE profiles are significantly associated with their physical activity levels. Specifically, R3STEP analysis indicated that lower physical activity levels increased the likelihood of being classified into the two low-efficacy profiles. A lack of physical activity demonstrated the strongest negative association with “Low Negative RESE,” followed by “Low Positive RESE,” and a comparatively smaller association with “Moderate RESE.” Research by Downs et al. demonstrated that physical activity significantly enhances emotion regulation self-efficacy (RESE) among high school students (Downs, 2014). Similarly, Yuan et al. found that physical activity promotes RESE in both college and middle school samples, with a particularly pronounced effect on the ‘regulation of negative emotions’ (Yuan et al., 2018). These findings are consistent with the overall results of the present study. According to Conservation of Resources (COR) theory, the academic competition and developmental pressures faced by high school students constitute a continuous depletion of resources (Hobfoll, 1989). Under such high-stress conditions, emotion regulation serves as a high-energy-consuming process of resource allocation. Without sufficient physical activity, individuals have fewer opportunities for recovery and positive experiences, such as building a sense of mastery or establishing regular routines. Consequently, this leaves them in a state of diminished energy and reduced psychological support (Hobfoll, 2001). In this situation, negative emotions become harder to suppress effectively, while positive emotions are more difficult to activate and sustain. This dual impairment leads to lower efficacy across positive and negative dimensions, ultimately manifesting as difficulty in emotion regulation.

In contrast, the “Proficient RESE” group demonstrates a superior advantage in resource reserves. Regular physical activity can be viewed as a long-term accumulation of psychological capital. Through physical activity, individuals gain a sense of belonging, efficacy experiences, and resilience (Jindo et al., 2018; Parschau et al., 2014), which together build a stable resource reservoir. When facing stressful challenges, these ample reserves enable individuals to invoke adaptive strategies, such as cognitive reappraisal. This process effectively blocks the excessive consumption of energy by negative emotions, thereby maintaining a state of high self-efficacy (Huahua et al., 2022; Xinyue, 2023).

Furthermore, this study found that the predictive effect of low physical activity on the “Low Negative RESE” profile (OR = 3.381) was significantly stronger than that on the “Low Positive RESE” profile (OR = 2.127). This differential association suggests a potential asymmetry in the degree to which positive and negative regulatory emotional self-efficacy rely on external resources. Integrating the Conservation of Resources (COR) theory, it can be inferred that regulating negative emotions, such as calming anger or frustration, typically requires a higher level of cognitive and executive control resources. Therefore, in the absence of physical activity as a potential channel for resource replenishment, these related regulatory functions may be more susceptible to impairment. Conversely, positive emotion regulation, because it is accompanied by pleasurable experiences, may generate a resource compensation effect to a certain extent, resulting in relatively less impairment (Hobfoll et al., 2015; Westman et al., 2004). Although the cross-sectional design of this study precludes a direct examination of the resource depletion mechanism, this asymmetrical predictive pattern provides a theoretical explanatory perspective for understanding how lifestyle factors differentially impact distinct emotion regulation dimensions. It also offers an empirical basis for future research to explore the developmental trajectories of adolescents’ emotional self-efficacy from the perspective of resource allocation and compensation mechanisms.

### 4.4 Limitations

This study has certain limitations. First, as our data collection focused solely on core demographic indicators, physical activity, and regulatory emotional self-efficacy indicators, it did not include measurements of variables such as socioeconomic status (SES) and academic stress; these unmeasured psychosocial factors may influence students’ regulatory emotional self-efficacy and may limit the comprehensiveness of the model. Future research could employ a more comprehensive design, incorporating these lifestyle, environmental, and psychological covariates, to validate and extend the current findings. Second, the use of self-report scales in this study is inevitably subject to social desirability bias, potentially exaggerating regulatory emotional self-efficacy that aligns with favorable stereotypes, causing the results to deviate from real data, and thereby masking the relationship between physical activity and regulatory emotional self-efficacy profiles. Third, this study adopts a cross-sectional design, which limits our ability to infer causal relationships between physical activity and regulatory emotional self-efficacy profiles. Future research could conduct long-term longitudinal studies to examine the stability of high school students’ regulatory emotional self-efficacy profiles and whether their relationship with physical activity changes over time.

## 5 Conclusion

1. There are four latent profiles of RESE among high school students: Low Negative Regulatory Emotional Self-Efficacy, Low Positive Regulatory Emotional Self-Efficacy, Moderate Regulatory Emotional Self-Efficacy, and Proficient Regulatory Emotional Self-Efficacy. Among these, the Low Positive Regulatory Emotional Self-Efficacy group is the most prevalent.
2. Gender significantly predicted high school students’ membership in latent profiles of RESE. Female students were more likely than male students to be classified into the Low Negative Regulatory Emotional Self-Efficacy group. Age and family structure showed no significant association with profile membership.
3. Physical activity is a significant predictor of latent profiles of RESE in high school students. Lower levels of physical activity were associated with the highest likelihood of being classified into the Low Negative RESE profile, followed by the Low Positive RESE profile. Individuals with high levels of physical activity had a higher probability of being classified into the Proficient RESE profile.

## Data Availability

The raw data supporting the conclusions of this article will be made available by the authors, without undue reservation.
