# Supplementary material for: Relationship between physical activity and latent profiles of regulatory emotional self-efficacy among high school students: a latent profile analysis
Source: Front Psychol. 2026 Mar 9;17:1796256. doi: 10.3389/fpsyg.2026.1796256 (PMC13006652; doi:10.3389/fpsyg.2026.1796256)
Supplement: Supplementary file 1 [file Table_1.DOCX]

Table S1. Descriptive Statistics of the Study Sample

| Variables |  | *M / N* | *SD / %* |
| --- | --- | --- | --- |
| Age |  | 16.46 | 0.71 |
| Gender | Male | 1205 | 53.1% |
|  | Female | 1064 | 46.9% |
| Family types | Intact family | 2106 | 92.8% |
|  | Blended family | 66 | 2.9% |
|  | Single-  parent family | 97 | 4.3% |
| Physical activity | Low physical activity | 504 | 22.2% |
|  | Moderate physical activity | 1095 | 48.3% |
|  | High physical activity | 670 | 29.5% |
| Regulatory Emotional Self-Efficacy | POS | 16.72 | 2.92 |
|  | DES | 14.22 | 3.86 |
|  | ANG | 13.75 | 4.02 |

Note: M = Mean, SD = Standard Deviation, N = Number of participants, % = Percentage. PA = Physical Activity; POS = Perceived Self-Efficacy in expressing positive affect; DES = Perceived Self-Efficacy in managing despondency/distress; ANG = Perceived Self-Efficacy in managing anger/irritation.
